# Efficacy of a systematic depression management program in high utilizers of primary care: a randomized trial

**DOI:** 10.1186/1472-6963-12-298

**Published:** 2012-09-03

**Authors:** Anne Berghöfer, Astrid Hartwich, Michael Bauer, Jürgen Unützer, Stefan N Willich, Andrea Pfennig

**Affiliations:** 1 Institute for Social Medicine, Epidemiology and Health Economics, |Charité University Medical Center, 10098, Berlin, Germany; 2 Department of Anesthesia and Intensive Care, Waldkrankenhaus Spandau, Berlin, Germany; 3 Department of Psychiatry and Psychotherapy, Carl Gustav Carus University Hospital, Technische Universität Dresden, Dresden, Germany; 4 Department of Psychiatry and Behavioral Sciences, School of Medicine, University of Washington, Seattle, WA, 98195, USA

## Abstract

**Background:**

Approximately 25% of so-called high utilizers of medical care are estimated to suffer from depression. A large proportion of these individuals remain undiagnosed and untreated. This study aims to examine the effects of a systematic screening and collaborative treatment program on depression severity in small primary care practices of the German outpatient health care system.

**Method:**

High utilizers of primary care who screened positive for depressive symptoms on the Brief Psychiatric Health Questionnaire (B-PHQ) were further diagnosed using the DIA-X, a standardized diagnostic interview, performed by trained and supervised interviewers. Patients with major depression were randomized (cluster randomization by practice) to (a) a six-month treatment program of pharmacotherapy, standardized patient and provider education, and physician and patient counseling or (b) six months of usual medical care. All subjects were followed for a 12-month observation period using the 17-item Hamilton Depression Rating scale (HAMD-17) rated by the treating physicians and the B-PHQ-9 rated by the patients.

**Results:**

A total of 63 high utilizer patients were included in the trial (17 male, 46 female), 19 randomized to intervention, 44 to usual care. The mean age was 49.7 (SD 13.8). Most patients had one or more somatic co-morbidities. There was no significant difference in response (defined as a decrease in the HAMD-17 sum score of at least 50%) after six months of treatment (50% vs. 42%, p = 0.961, all analyses adjusted for age) and after 12 months of treatment (83% vs. 54%, p = 0.282) between groups. Using patient self-rating assessments with the B-PHQ-9 questionnaire the intervention was superior to treatment as usual at six months (83% vs. 16%, p = 0.000).

There was no significant difference in HAMD-17 depression severity at six months between the groups (10.5 (SD 7.6) vs. 12.3 (SD 7.8), p = 0.718), but a trend at 12 months (4.7 (SD 8.0) vs. 11.2 (SD 7.4), p = 0.083). Again, using B-PHQ-9 sum scores depression severity was significantly lower in the intervention group than in the treatment as usual group after six months (6.4 (SD 5.2) vs. 11.5 (SD 5.8), p = 0.020), but not at 12 months (7.9 (SD 8.7) vs. 9.0 (SD 5.2), p = 0.858).

**Conclusion:**

A systematic collaborating treatment program for depression in high utilizers in primary care showed superiority to treatment as usual only in terms of patientsÂ´ self-assessment but not according to physiciansÂ´ assessment. The advance of the intervention group at 6 months was lost after 12 months of follow-up. Overall, positive results from similar trials in the US health care systems could not be confirmed in a German primary care setting.

## Background

Depression is regarded as one of the leading causes of disability worldwide [1]. Within the World Mental Health Survey Initiative a 12-month prevalence of major depressive disorders of 5.5% in high-income countries and 5.9% in middle and low income countries was reported [2]. The pan-European survey of depression across six countries DEPRES detected a 6-month prevalence of 17% [3]. The more recent ESEMeD project reported a lifetime prevalence of major depression of 12.8% [4]. Comparable prevalence rates for depression were found by the German National Health Interview and Examination Survey (GHS), a government mandated nationwide study including a random sample drawn from population registries [5].

Those seeking help mostly consult facilities in primary care. However, depression in primary care is often overlooked and severely undertreated. DEPRES II demonstrated that 70% of depressed patients had received no antidepressant therapy during their most recent depressive episode [6,7]. In the German GHS sample, only approximately 50% of patients with any depressive disorder received at least minimal intervention [5]. The Depression 2000 study, which included 20,421 patients in 633 primary care practices, detected a point prevalence of depression of 11% in German primary care practices. Physicians recognized 59% of all ICD 10 depression diagnoses correctly, however, false diagnoses were given in 11.7% [8].

Unrecognized or undertreated depression is especially problematic in patients who make frequent use of healthcare services and are commonly referred to as “high utilizers” in health-systems research. Depression is thought to increase the use of medical care [9], and approximately 25% of high utilizers are estimated to suffer from depression [10,11].

With these figures in mind, primary care-based management programs would seem useful in improving the care of depressed patients, and have been proposed by a range of international experts [12,13]. Modern care-management programs are multifaceted and generally incorporate a systematic approach to treatment (e.g. evidence-based practice guidelines or algorithm-guided treatment regimens); patient and family education programs; practice reorganization to meet the needs of chronically ill patients; and available expert consultation for practitioners and staff, and care-manager for tracking patient’s visits and enhancing compliance. More than 40 studies have proven the efficacy of various combinations of these facets during the last 2 decades [14-17].

Several of these studies focus on applying treatment algorithms within inpatient and/or outpatient psychiatric settings, e.g. Texas Medication Algorithm Project, TMAP [18], German Algorithm Project, GAP [19], both in psychiatric settings, and Sequenced Treatment Alternatives to Relieve Depression STAR*D in primary care [20], (see [21] for a comprehensive review).

Only a few studies of collaborative care have included a systematic treatment algorithm for depression in primary care. The IMPACT (Improving Mood: Promoting Access to Collaborative Treatment) trial [22-24] and the PROSPECT study [25] focused on elderly and geriatric patients. The trial of Ijff et al was conducted in the Netherlands, combining multifaceted collaborative care elements in small primary care practices [26]. Controlled studies on depression care management programs designed specifically to optimize the treatment of depression in high utilizers are rare [27].

Most of the collaborative care studies were performed in the US within health plans, large primary care clinics or in collaboration with large insurance funds, which allowed for providing additional staff for care management and resorting to existing coordinating structures. Other studies were performed within the UK health system, a national health system with top down regulation and primary care system with gate keeping function.

The results cannot be easily transferred to the German health system. Outpatient care is delivered by physicians working alone or in small cooperatives, primary care practices generally do not have gatekeeper function. The previous fee-for-service reimbursement has increasingly been replaced by lump-sums for various basic services and is capped. Incentives for collaboration and improving quality of care are lacking [28].

The only controlled trial on collaborative primary care in Germany performed by Gensichen and coworkers showed superiority of a systematic screening and monitoring of patients for depression and a care management with regular telephone contacts by trained practice staff (PRoMPT) in reducing depression symptoms [29].

While the physicians in PRoMPT were free with regard to their treatment decisions, we conducted a study which combined an antidepressant treatment algorithm with a collaborative care concept including systematic screening, monitoring and care management by practice staff as well as expert supervision and assistance with referral which can be implemented within the German outpatient health care system.

## Methods

**Figure 1:**
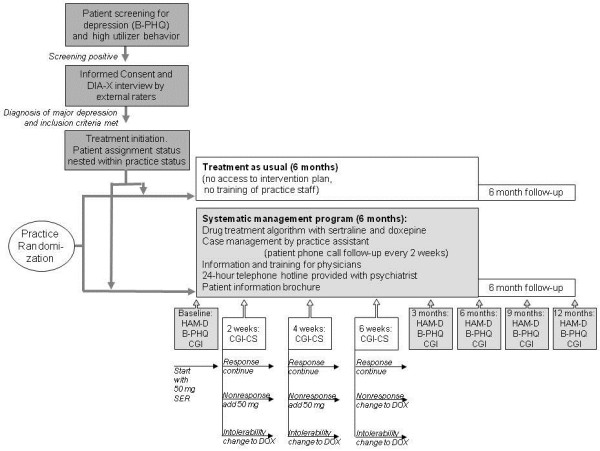
Study design of screening and randomized treatment of patients with depression in primary care (B-PHQ = Brief Patient Health Questionnaire, DIA-X = Diagnostic Expert System for Psychiatric Disorders, HAMD = Hamilton Depression Scale, CGI = Clinical Global Impression Scale, CGI-CS = Clinical Global Impression Change Score, SER = Sertralin, DOX = Doxepine).

A total of 31 primary care physicians in Berlin and Brandenburg participated in the present study which was performed between 2004 and 2005. Primary care practices were assigned randomly to implement a systematic depression-management program or to provide treatment as usual for a period of six months and a subsequent follow-up period of six months. The study design is illustrated in Figure 1.

### Inclusion and exclusion criteria

To be included in the study, patients had to:

(1) be classified as a high utilizer of healthcare (≥5 visits to any physician within the most recent completed quarter of the year, including physician and patient initiated appointments)

(2) screen positive for depression (i.e. have at least four positive answers on the Brief Patient Health Questionnaire (B-PHQ) including the symptom of feeling down and/or little interest or pleasure in doing things)

(3) be diagnosed with unipolar depressive disorder and be experiencing a major depressive episode of at least moderate severity according to the Diagnostic Expert System for Psychiatric Disorders (DIA-X)

(4) be at least 18 years old

(5) have sufficient cognitive ability and language skills to participate in the study

(6) and provide written, informed consent.

Patients were excluded from participation in the study if they

(1) had already received treatment for their current depressive episode with antidepressants or formal psychotherapy

(2) were currently experiencing a minor depressive episode according to the DIA-X

(3) were diagnosed with a psychiatric disorder other than unipolar depressive disorder (e.g. bipolar disorder, schizophrenia, schizoaffective disorder, alcohol and/or drug dependence)

(4) exhibited suicidal behavior on more than half of the days during the two weeks before the day on which they completed the B-PHQ (item on the B-PHQ)

(5) had contraindications to sertraline

(6) or were judged by the attending physician to be unable to participate due to severe medical illness.

### Randomization

Practices who agreed to participate in the study were assigned using computer-based randomization in the study coordinating center either to the intervention arm or to the treatment as usual arm. Patient random assignment status was nested within the practice status. Practice staff was not blinded to the allocation to a group due to the necessary training in the intervention group, however, treatment as usual practices did not have access to the provided intervention plan.

### Sample recruitment

High utilizers of healthcare services were identified by staff members in each study practice using either their computerized patient documentation system or the questionnaire employed to screen patients for participation in the study. Subsequently, patients were evaluated for depression using the B-PHQ, a short self-report instrument extracted from the PRIME-MD Patient Health Questionnaire [30,31]. With a cut-off of at least four positive answers including the cores symptoms, sensitivity is 71% and specificity 91% [32]. High utilizers who screened positive for depression were asked for informed consent by their attending physician. After providing written informed consent, patients’ contact data were transmitted to the study coordinating center. Diagnoses were validated at the coordinating center by trained and supervised interviewers using the DIA-X, a standardized diagnostic telephone interview [33-35]. If the patient met all inclusion criteria the coordinating center informed the attending primary care physicians to initiate the first study visit; if the patient was excluded, a consulting psychiatrist (AP) at the coordinating center made recommendations regarding patient referrals (e.g. to a private-practice psychiatrist or a hospital).

### Intervention program

The intervention program included three categories of interventions: (1) Elements focusing on the primary care practice, (2) elements focusing on the participating physicians, and (3) elements focusing on patients and lasted six months followed by a follow up period of another six months. During the second six months physicians could either continue the treatment according to the systematic depression-management program or change to any other treatment or stop treatment.

The primary care practice was provided with a treatment algorithm applying an antidepressant (sertraline or in case of insufficient efficacy or intolerability, doxepine) and received simple and efficient instruments to assess depression severity and course (B-PHQ). In addition, the office staff was trained in using the intervention program and applying the case management via telephone. The participating physicians were informed and trained in using the instruments and applying the intervention program and received a compact information brochure. At any time they had access to a telephone hotline staffed by a psychiatric consultant to help answer any questions the primary care physicians might have.

The patients received an information brochure meeting the needs of the patients and their relatives. The material included information about causes, symptoms and course of depression, step by step self-help recommendations which should encourage patients to participate in activities. Patients’ feedback regarding the recommendations in the brochure were not included in the study.

The antidepressant treatment algorithm was performed as shown in Figure 1. Insufficient response was defined as a CGI change of condition score < 4. In case of nonresponse or intolerance to doxepine the patient left the study and was referred to a mental health specialist. Sertraline was provided to the primary care physician free of charge for up to six months; doxepine had to be prescribed according to standard billing procedures. Doxepine was chosen to ensure that second-line treatment would involve an antidepressant with a different mechanism of action. Moreover, doxepine is well known to primary care physicians in Germany and does not overextend their limited budget for prescription medication.

Case-management consisted of structured telephone calls with the patient conducted by a trained staff member from the primary care practice. During the calls, patients were asked if they had a sufficient supply of medication and if they had experienced any side effects. Patients were also reminded about their next visit to the primary care practice. The calls were planned to last for approximately five to ten minutes each and were performed by the same practice staff member.

### Treatment as usual

Physicians who were randomized to the non-intervention group were asked to treat their patients as they normally would do for this diagnosis. The physicians in this group were free to choose any treatment they felt was necessary during the first six months. During the follow-up period of months seven to 12 they could either continue or stop their treatment as usual.

### Assessment instruments and endpoints

Participating patients were assessed at the beginning of the study and after three, six, nine, and 12 months. In the intervention arm, patients were seen additionally every two weeks following study entry until the optimal drug and dosage were found. Structured assessment instruments used were the B-PHQ, Clinical Global Impressions (CGI) [36], and Hamilton Depression Rating Scale (HAM-D, 17-item version [37], the last being guided by a structured interview [38]. Additionally, the CRF included questions on comorbid somatic illnesses, use of medications (including non-psychotropic’s), and the use of healthcare services. All visits and assessments were performed by the treating physician in order to avoid any interrater variation in the assessment or disruption in the treatment process.

The primary endpoint was the response (defined as a decrease in the HAMD-17 sum score of at least 50%) in physicians’ assessment after 6 months of treatment.

Secondary endpoints were the response in physicians’ assessment after 12 months of treatment, the response (defined as a decrease in the sum score of at least 50%) in patient self-assessment with the B-PHQ-9 questionnaire after 6 and 12 months, and the depression severity measured by both the HAMD-17 and the B-PHQ-9 after 6 and 12 months.

### Sample size calculation

The sample size calculation was based on results of studies of sertraline in primary care (e.g. [39]). It was assumed that 80% of patients in the intervention group would respond according to the HAMD after six months to treatment with sertraline and 50% would respond to treatment as usual after 6 months in the non-intervention arm of the study. With the alpha error set at 0.05 and the beta error at 0.2, the sample size was projected to be 37 patients per group (i.e. a total of 74 patients). The difference in the percentage of responders was tested using a one-sided chi-square test.

Because of the cluster-randomization (not patients but practices will be randomized), the sample size was adjusted using a variance inflation factor. With 15 clusters including five patients each (in total 75 patients) and a variance inflation factor of 1.16 a sample size of 88 will be needed.

### Analysis

All analyses were based on an intention-to-treat approach. Apart from complete subject analysis, analyses with missing data replaced by last observation carried forward (LOCF) were done. Comparability of the groups at baseline was assessed using *t* tests and chi-square tests. To identify influencing variables, linear regression analyses with age, gender, education, treatment group, and depression severity at baseline were used. One-sided chi-square-test and ordinal regression analysis (including influencing variables at baseline as covariates) were used to analyze categorical data, ANCOVA were applied to analyze group differences of metric data including variables different between the groups at baseline as covariates. ANCOVA for repeated measurements were used to investigate differences in the course of treatment from baseline over three, six, nine, and 12 months between the groups, again including variables differing between the groups at baseline as covariates.

The study protocol was approved by the local University Ethics Board (Charité - Universitätsmedizin Berlin, Germany) and adheres to the Declaration of Helsinki. All patients gave written informed consent before participating in the study.

## Results

### Baseline characteristics of the patients

A total of 129 high utilizers were screened positive with depressive symptoms. Of these, 63 patients were included in the study. Various reasons for exclusion and baseline characteristics of the patient sample are reported in Table 1. Most of the study patients were of working age; a substantial proportion was unemployed and had a household income equivalent of lower than US$ 1000. A large percentage of patients had a somatic comorbidity and were on sick leave. The intervention and usual-treatment groups differed only with respect to age, therefore statistical analyses were adjusted for age.

**Table 1:** Baseline characteristics of the patients included in trial

High utilizer screened with major depression in the B-PHQ			129
• DIA-X negative for psychiatric disorder			7
• Other non-affective disorder (psychosis, somatisation, etc.)			11
• DIA-X positive for unipolar major depression and study entry			63
• Excluded from study participation			48
- depressive episode already remitted or treated			10
- dysthymia			15
- inadequate cognitive or language ability to complete questionnaires			4
- withdrawal of informed consent			1
- severe suicidal ideation			18
	**Intervention n = 19**	**Treatment as usual n = 44**	**p-value**
Age (years, mean ± SD)	44.5 ± 12.0	51.9 ± 14.0	0.048
Gender (% male)	21	30	0.552
Education			0.737
- % no degree	12	5	
- % elementary school	29	28	
- % secondary school	35	35	
- % A-levels	18	15	
- % university degree	6	18	
Work status			0.787
- % on sick leave	47	55	
- % unemployed	26	20	
- % retired	16	18	
% with household income equivalent < US$ 1000	42	32	0.553
Depression severity (HAMD sum score, mean ± SD)	20.6 ± 4.4	18.5 ± 6.5	0.247
Self-rated depression (B-PHQ sum score, mean ± SD)	17.6 ± 4.4	16.0 ± 3.7	0.089
% with somatic comorbidity			
- cardiovascular disease	84	61	0.934
- diabetes	11	9	0.843
- chronic pain syndrome	63	50	0.272

Twenty of the patients in the as usual treatment arm (45.5%) received psychopharmacological therapy (one with amitriptyline, eight with an SSRI, five with mirtazepine, one with a combination of amitriptyline and mirtazepine, one with a combination of trimipramine and opipramol, one with venlafaxine, and four with St. John’s wort). In the seven patients who were treated with psychotherapy, five also received antidepressants. Fifteen patients (34.1%) did not receive any antidepressive treatment. For six patients in the usual treatment arm, no treatment information was available.

Thirteen of the patients in the intervention arm of the study were treated with sertraline (68.4%). One patient was treated with doxepine (in addition to psychotherapy). Two patients did not receive any antidepressive treatment. No treatment information was available for three patients in the intervention arm.

### Study endpoints

After six months as well as after 12 months of treatment both patients in the intervention and in the treatment as usual group showed response but groups did not differ according to physicians’ assessment (see Table 2). Replacing missings with LOCF did not change the results. The effect size for response after 6 months was 1.4 (0.4 - 5.1), after 12 months 4.2 (0.4 - 39.9).

**Table 2:** Treatment response (defined as 50% change from baseline sum score) in physicians’ assessment using HAMD-17 item scale and by patients’ self-assessment using B-PHQ 9 questionnaire, and HAMD-17 sum scores and B-PHQ 9 sum scores at 6 month and at 12 month in the intervention group and in the group treated as usual, all analyses adjusted for age

	**Intervention**	**Treatment as usual**	**p-value**
*Physicians’ assessment*			
Response in HAMD-17 score at 6 months (n, %), n = 45	6 (50)	14 (42)	0.961
Missing values replaced by LOCF, n = 57	6 (38)	15 (37)	0.756
Response in HAMD-17 score at 12 months (n, %), n = 41	5 (83)	19 (54)	0.282
Missing values replaced by LOCF, n = 59	6 (38)	20 (47)	0.388
Depression severity by HAMD-17 score at 6 months (mean, SD), n = 45	10.5 (7.6)	12.3 (7.8)	0.718
Missing values replaced by LOCF, n = 58	12.8 (7.7)	13.8 (8.3)	0.954
Depression severity by HAMD-17 score at 12 months (mean, SD), n = 41	4.7 (8.0)	11.2 (7.4)	0.083
Missing values replaced by LOCF, n = 60	11.5 (8.1)	12.8 (8.2)	0.847
*Patients’ self assessment*			
Response in B-PHQ 9 score at 6 months (n, %), n = 44	10 (83)	5 (16)	0.000
Missing values replaced by LOCF, n = 60	10 (56)	7 (17)	0.002
Response in B-PHQ 9 score at 12 months (n, %), n = 38	4 (67)	15 (47)	0.346
Missing values replaced by LOCF, n = 60	7 (37)	19 (43)	0.887
Depression severity by B-PHQ 9 score at 6 months (mean, SD), n = 45	6.4 (5.2)	11.5 (5.8)	0.020
Missing values replaced by LOCF, n = 60	9.8 (6.9)	11.7 (5.4)	0.375
Depression severity by B-PHQ 9 score at 12 months (mean, SD), n = 42	7.9 (8.7)	9.0 (5.2)	0.858
Missing values replaced by LOCF, n = 60	10.6 (7.5)	9.6 (5.1)	0.428

Depression severity as evaluated by physicians using the HAMD-17 scale at six months did not differ between the groups. At 12 months depression severity in the intervention group tended to be lower than in the usual treatment group as measured using the HAMD-17 scale. However, at all time points after baseline the scores were numerically lower in the intervention group. Replacing missing data by the last observation did not change the results.

Using patients’ self assessment with the BPHQ-9 questionnaire the intervention group yielded superior results (at least 50% reduction of the baseline BPHQ-9 sum score) after six months of treatment but not at 12 months (see Table 2). Replacing missing data by the last observation did not change results. The effect size for response after 6 months was 27.0 (4.5 - 162.2), after 12 months 2.3 (0.4 - 14.2).

When analyzing the sum scores for self-rated depression using the B-PHQ-9, patients had significantly lower sum scores in the intervention group than in the as usual group at six months. However, at 12 months no significant difference between the groups could be observed (see Table 2). Again, at all time points after baseline the scores were numerically lower in the intervention group.

When replacing missing data by the last observation the difference at six months was not any longer statistically significant. Without replacement, there was no difference in the score at 12 months.

## Discussion

In the present study, a substantial number of patients classified as high utilizers of healthcare services and diagnosed with major depression could be recruited for antidepressant treatment. The systematic depression-management program was shown to be superior to treatment as usual in primary care after 6 months according to patient self-rating of depression. However, severity of depression at six months measured by physicians’ assessment using the HAMD scale – the primary endpoint of the study – did not differ between the two groups. In short, the depression-management program was superior only with respect to the secondary endpoint patient self-assessment.

Our study differed in so far from various other controlled trials of multifaceted interventions to improve depression in primary care in that we used a formal medication algorithm which facilitates the prescription of antidepressant medication for the primary care physician. Against the background of an extensive range of antidepressant products and complex recommendations on dose and length of treatment this might have lowered the threshold to treat depression in primary care. In addition, our intervention plan was designed to use existing primary care office staff as opposed to most primary care collaborative care trials that introduced mental health trained staff into the office. Using primary care office staff on the one hand facilitated the long-term implementation of multifaceted interventions after the clinical trial had stopped. On the other hand, this reflected the routine conditions in primary care more appropriately and enhanced the external validity of the results.

Our study was performed in small primary care practices which are typical for the German outpatient health care system, not possessing routine pathways for referral and networking and leading to substantial waiting time when referring to a specialist. In this regard our study was designed to be feasible for routine primary care.

Fourthly, this study focused on high utilizers who represent a high risk group for depressive symptoms and thus mean a substantial economic burden for the health care system.

Compared to the large collaborative care trials in the UK and U.S. [14-17] the difference in response to depression treatment between intervention and usual care was modest. The outcome of the intervention group was not persistently superior to usual care. The findings of our study have to be evaluated with several limiting factors kept in mind. Firstly, the number of patients ultimately included in the study did not reach the number projected in the sample size calculation. As a result, the study was slightly underpowered despite the various measures that were taken to encourage the recruitment of study patients. The number of primary care physicians invited for participation in the study was increased during the recruitment period to the final number of 1,725 physicians in Berlin and surroundings in order to find more study sites. Of these, only 62 physicians agreed to participate in the study. Because some primary care physicians were not able to identify high utilizers using their particular computerized patient documentation system, patients could also be identified using charts or asking the patients themselves. Because most practices had difficulties screening patients with the provided material, screening was conducted by trained doctoral students. The reimbursement to the study sites for screening more patients was increased. By this means we tried to increase the number of patients which were eligible for study entry.

The data suggest that there were quite substantial differences in scores that persist over the course of the study. We assume that if the study would have had sufficient power, there would likely have been clinical and also statistical significant differences between both groups.

Secondly, patients in the intervention arm showed various divergences from the study design. Only two-thirds of the 19 patients included in the study were in fact treated with sertraline, the first-choice drug in our systematic disease-manage program. One patient received doxepine due to intolerance to sertraline, and five patients did not receive any pharmacological treatment or data were missing due to a lack of documentation in the primary care practice. In the treatment-as-usual group, approximately half of the patients received some form of antidepressant treatment. We were surprised about the high rate of treatment in usual care and the range of treatments used. However, with the treatment-as-usual arm not being as undertreated as we had expected, the difference between the percentage of treated patients in the two study arms became small.

Thirdly, the intervention group and the usual-treatment group were of different size. This was due to the necessary randomization of physician practices as a whole rather than single patients. The latter was not possible because patients randomized to different study arms within the same practice would have lead to contamination of treatment effect. Indeed cluster randomization was practiced in nearly all of the collaborative care trials. During the study some physicians were more active in recruiting patients, which ultimately resulted in different group sizes.

We used doxepine as the antidepressant of second choice for two reasons. Firstly, if during the treatment period sertraline had to be stopped due to adverse events or nonresponsive, the treating physician should have been able to change to an antidepressant which has a different mechanism of antidepressive action. Although the switch from one antidepressant to another in non responders is discussed controversially there is evidence for switching from an SSRI to a tricyclic drug [40,41]. Secondly, doxepine is a well known antidepressant in primary care in Germany and does not burden the limited budget of the GPs for prescriptions which is around 15 Euro per patient per month in the region of the study sites. The comparably expensive sertraline was distributed as a study medication for free, whereas the switch medication had to be prescribed by the physician. In order to avoid early treatment termination of sertraline due to the low prescription cap in practices this model was chosen.

The study did not include a follow-up of non responders after leaving the treatment plan. Patients and primary care physicians have freedom to choose a specialist for referral in the German health care system. Contracting new study sites during the ongoing study would have been unfeasible.

The systematic treatment plan provided the Clinical Global Impression Scale for diagnosing partial or nonresponsive during the treatment period. This comparably rough instrument was chosen to facilitate the study conditions for the GPs as far as possible. Using the HAMD scale as a basis for treatment decisions would have overstrained the willingness of the GP to participate in the study. On the other hand, using the CGI as a basis for treatment decisions might have been an inaccurate and imprecise approach and would have lead to early switches from sertraline to doxepine or to early fixation of the antidepressant dose. In addition, the assessment of depression severity using the HAMD and the CGI was done by the treating physician himself or herself and not by independent raters blind to the intervention status.

The discrepancy between the results measured by physicians’ rating using the HAMD-17 scale and the patients’ self rating with the B-PHQ-9 questionnaire is striking. This may have simply been the effect of the small sample size. However, although the HAMD scale served as the gold standard, it may have had limited feasibility in primary care due to its length and the leeway which is given to the raters to score the patients’ answers. We applied the HAMD scale together with a standardized interview and a comprehensive guideline for scoring, and physicians performed a rater training before study start. Not all physicians might have used the material properly or pressure of time in routine care might have hampered the proper application.

Self-rating measures were used as a primary outcome in many of the large collaborative care studies [22,42,43] and have been shown to provide valid ratings in non-psychotic depressed outpatients [44]. The B-PHQ-9 has been proven as a valid instrument for measuring treatment efficacy and the standard definition of improvement agreed sufficiently with other definitions of patients’ improvement [45,46]. Based on the B-PHQ-9 scores our study results were comparable to those of other collaborative care trials.

While most of the larger trials were using additional staff for care-management, embedding additional counseling or psychotherapeutic approaches or working with more complex medication algorithms, which might not work without additional funding or support by academic centers, our systematic depression management program was mainly designed to fit into routine primary care in the German outpatient health care system, based on using the available resources and being appropriate for long-term use. However, the adherence to the medication algorithm was not complete and frequency of using the supervision by psychiatric experts was altogether low. Embedded in daily routine of the primary care practice doctors might have been overstrained by the management program.

A number of barriers to adequate depression management in Germany have been discussed in the existing literature, and several of them may help explain the experiences described in this study. These include system-level barriers, such as practice organization, the fiscal separation of physical and psychiatric services by the Regional Associations of Social Health Insurance-accredited Physicians, and the subsequent lack of additional reimbursement for the increased costs associated with the care of psychiatric patients [47]. We tried to compensate by reimbursing the additional time spent on treating psychiatric patients. However, our financial resources were not enough to provide sufficient incentive.

## Conclusion

The study has shown moderate superiority of a systematic depression treatment and management program over a treatment as usual in primary care only from the patients’ perspective. The study gives limited indication that the utilization of systematic treatment plans for psychiatric disorders in small primary care practices in the German health system seems feasible and effective.

## Competing interests

The study was supported by Pfizer GmbH, Karlsruhe, by a limited grant and by supplying sertraline medication for intervention practices.

Beyond this the authors did not receive reimbursement, fees, funding, or salary from neither hold stocks or shares of an organization that may in any way gain or lose financially from the publication of this manuscript. They do not have any other financial or non-financial competing interests relevant to the subject of this article to declare.

## Authors’ contributions

AB and AP have made substantial contributions to study conception and design, acquisition of data, analysis and interpretation of data and drafting of the manuscript. AH has been involved in study conception, acquisition and analysis of data. MB, JU and SW have been involved in study conception and drafting the manuscript. All authors read and approved the final manuscript.

## Pre-publication history

The pre-publication history for this paper can be accessed here:

http://www.biomedcentral.com/1472-6963/12/298/prepub
